# Crystal structure of (1*Z*,2*E*)-cinnamaldehyde oxime

**DOI:** 10.1107/S2056989015023853

**Published:** 2015-12-16

**Authors:** Bernhard Bugenhagen, Nuha Al Soom, Yosef Al Jasem, Thies Thiemann

**Affiliations:** a Institute of Inorganic Chemistry, University of Hamburg, Hamburg, Germany; b Department of Chemistry, United Arab Emirates University, AL Ain, Abu Dhabi, United Arab Emirates; c Department of Chemical Engineering, United Arab Emirates University, AL Ain, Abu Dhabi, United Arab Emirates

**Keywords:** crystal structure, cinnamaldehyde, oxime, conformers, O—H⋯N hydrogen bonding, C—H⋯π inter­actions

## Abstract

The title compound, C_9_H_9_NO, crystallized with two independent mol­ecules (*A* and *B*) in the asymmetric unit. The conformation of the two mol­ecules differs slightly with the phenyl ring in mol­ecule *A*, forming a dihedral angle of 15.38 (12)° with the oxime group (O—N=C), compared to the corresponding angle of 26.29 (11)° in mol­ecule *B*. In the crystal, the *A* and *B* mol­ecules are linked head-to-head by O—H⋯N hydrogen bonds, forming –*A*–*B*–*A*–*B*– zigzag chains along [010]. Within the chains and between neighbouring chains there are C—H⋯π inter­actions present, forming a three-dimensional structure.

## Related literature   

For the other methods of preparation of the title compound, see: Mirjafari *et al.* (2011 ▸); Kitahara *et al.* (2008 ▸). For the uses of a such compound, see: Narsaiah & Nagaiah (2004 ▸); Jasem *et al.* (2014 ▸); Garton *et al.* (2010 ▸); Patil *et al.* (2012 ▸); Kaur *et al.* (2006 ▸); Boruah & Konwar (2012 ▸).
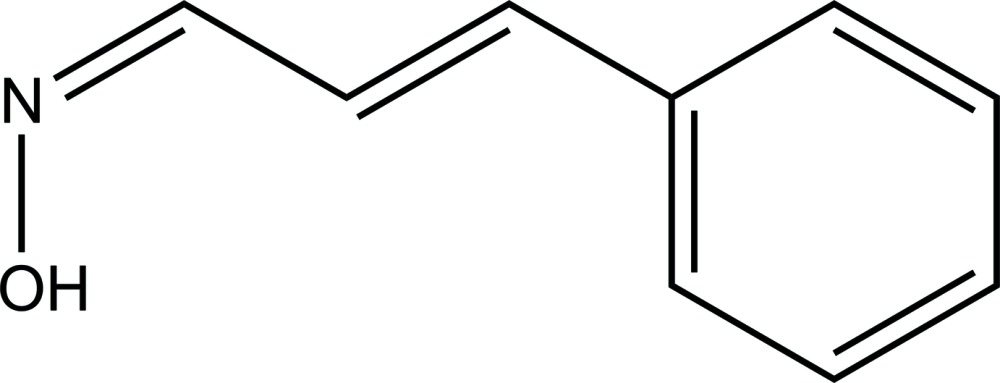


## Experimental   

### Crystal data  

C_9_H_9_NO
*M* *_r_* = 147.17
Orthorhombic, 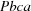

*a* = 10.231 (5) Å
*b* = 7.584 (3) Å
*c* = 41.816 (18) Å
*V* = 3245 (2) Å^3^
*Z* = 16
Mo *K*α radiation
μ = 0.08 mm^−1^
*T* = 100 K
0.2 × 0.2 × 0.1 mm

### Data collection  

Bruker APEXII CCD diffractometer
Absorption correction: multi-scan (*SADABS*; Bruker, 2013 ▸) *T*_min_ = 0.666, *T*_max_ = 0.746
34431 measured reflections
3944 independent reflections
3724 reflections with *I* > 2σ(*I*)
*R*_int_ = 0.022

### Refinement  

*R*[*F* ^2^ > 2σ(*F* ^2^)] = 0.043
*wR*(*F* ^2^) = 0.113
*S* = 1.10
3944 reflections
207 parameters
H atoms treated by a mixture of independent and constrained refinement
Δρ_max_ = 0.45 e Å^−3^
Δρ_min_ = −0.19 e Å^−3^

### 

Data collection: *APEX2* (Bruker, 2013 ▸); cell refinement: *SAINT* (Bruker, 2013 ▸); data reduction: *SAINT*; program(s) used to solve structure: *SHELXS97* (Sheldrick, 2008 ▸); program(s) used to refine structure: *SHELXL2014* (Sheldrick, 2015 ▸); molecular graphics: *PLATON* (Spek, 2009 ▸) and *Mercury* (Macrae *et al.*, 2008 ▸); software used to prepare material for publication: *OLEX2* (Dolomanov *et al.*, 2009 ▸).

## Supplementary Material

Crystal structure: contains datablock(s) I. DOI: 10.1107/S2056989015023853/su5260sup1.cif

Structure factors: contains datablock(s) I. DOI: 10.1107/S2056989015023853/su5260Isup2.hkl

Supporting information file. DOI: 10.1107/S2056989015023853/su5260Isup3.cml

**Fig. 1.**
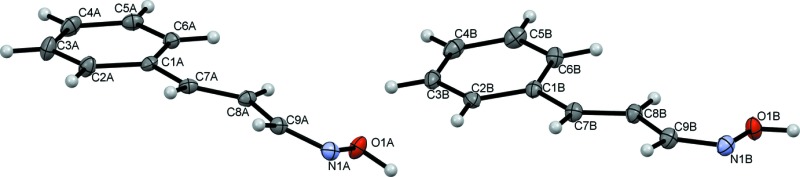
A view of the molecular structure of the two independent molecules (A and B) of the title compound, with atom labelling. Displacement ellipsoids are drawn at the 50% probability level.

**Fig. 2.**
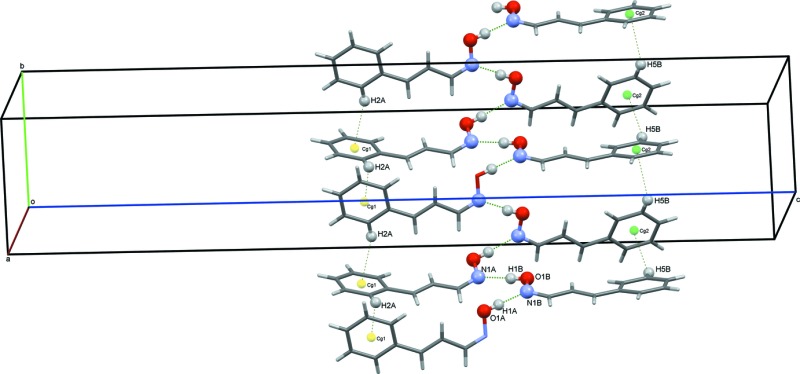
A partial view along the a axis of the crystal packing of the title compound. The O—H···N hydrogen bonds, and the C—H···π contacts between adjacent molecules are shown as dashed lines (see Table 1).

**Fig. 3.**
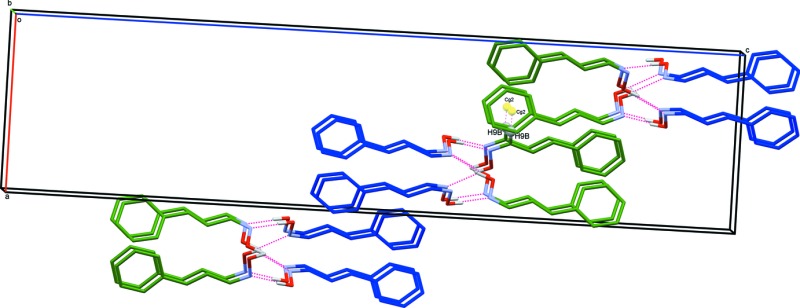
A view along the b axis of three stacked molecular motifs made of A (blue) and B (green) interconnected molecules forming chains along the b axis. The hydrogen bonds and C—H···π interactions are shown as dashed lines (see Table 1).

A B . DOI: 10.1107/S2056989015023853/su5260fig1.tif
A view of the mol­ecular structure of the two independent mol­ecules (*A* and *B*) of the title compound, with atom labelling. Displacement ellipsoids are drawn at the 50% probability level.

a . DOI: 10.1107/S2056989015023853/su5260fig2.tif
A partial view along the *a* axis of the crystal packing of the title compound. The O—H⋯N hydrogen bonds, and the C—H⋯π contacts between adjacent mol­ecules are shown as dashed lines (see Table 1).

b b . DOI: 10.1107/S2056989015023853/su5260fig3.tif
A view along the *b* axis of three stacked mol­ecular motifs made of A (blue) and B (green) inter­connected mol­ecules forming chains along the *b* axis. The hydrogen bonds and C—H⋯π inter­actions are shown as dashed lines (see Table 1).

CCDC reference: 1441984

Additional supporting information:  crystallographic information; 3D view; checkCIF report

## Figures and Tables

**Table 1:** Hydrogen-bond geometry (Å, °) *Cg*1 and *Cg*2 are the centroids of rings C1*A*–C6*A* and C1*B*–C6*B*, respectively.

*D*—H⋯*A*	*D*—H	H⋯*A*	*D*⋯*A*	*D*—H⋯*A*
O1*A*—H1*A*⋯N1*B* ^i^	0.91 (2)	1.85 (2)	2.755 (2)	174 (2)
O1*B*—H1*B*⋯N1*A* ^ii^	0.92 (2)	1.95 (2)	2.853 (2)	170 (2)
C2*A*—H2*A*⋯*Cg*1^iii^	0.95	2.70	3.563 (2)	151
C5*B*—H5*B*⋯*Cg*2^iv^	0.95	2.80	3.508 (2)	132
C9*B*—H9*B*⋯*Cg*2^v^	0.95	2.82	3.717 (2)	159

Symmetry codes: (i) 
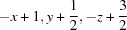
; (ii) 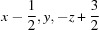; (iii) 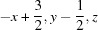; (iv) 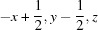; (v) 
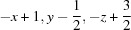
.

## supplementary crystallographic information

### S1. Structural commentary

Many uses of cinnamaldehyde oxime have been reported, such as the conversion to cinnamo­nitrile (Narsaiah & Nagaiah 2004; Jasem *et al.* 2014), conversion to cinnamide (Garton *et al.* 2010), and as a starting material for *N*-heterocycles: tetra­zole (Patil *et al.* 2012), isoxazoline (Kaur *et al.* 2006), and izoxazoline (Boruah & Konwar 2012).

The title compound, crystallized with two independent molecules A and B in the asymmetric unit (Fig. 1). The aromatic ring in molecule A (C1A—C6A) forms a dihedral angle of 15.38 (12)° with the oxime group (C9A/N1A/O1A), compared to a corresponding angle of 26.29 (11)° in molecule B. This conformational difference between molecules A and B is due to bond rotation, not only about bonds (C1—C7) and (C8—C9) but also of that of (C7—C8), where in molecule A the torsion angle C1—C7—C8—C9 is −174.32 (11)° while in molecule B the corresponding angle is −179.24 (11) °. The bond lengths, C7—C8, of molecules A and B are similar.

In the crystal, the A molecules align opposite B molecules, and they are linked *via* O—H···N hydrogen bonds forming –A—B—A—B– zigzag chains propagating along the *b* axis (Table 1 and Fig. 2). Adjacent molecules of the same type are tilted against each other, with the aromatic rings (C1—C6) being inclined to one another by 77.64 (2) and 59.04 (2)° for molecules A and B, respectively. In addition, adjacent molecules of the same type exhibit weak C—H..π (C2A—H2A···C*g*1 and C5B—H5B···C*g*2) contacts along the *b* axis direction (Table 1 and Fig. 2). Along the *c* axis, inversion related dimers stack with an offset of 11.47 (2) Å and connected *via* a weak C—H..π (C9B—H9B···C*g*2) contact (Fig. 3 and Table 1).

### S2. Synthesis and crystallization

To a solution of cinnamaldehyde (1.32 g, 10 mmol) in ethanol (20 ml) was added drop wise a solution of hydroxyl­amine hydro­chloride (2.74 g, 39.7 mmol) in water (7.5 ml), and the resulting mixture was stirred at 60 ^*o*^C for 3 h. Thereafter, about half of the solvent was removed *in vacuo*, and the remaining reaction mixture was poured into water (50 ml) and extracted with CHCl_3_ (3 × 20 ml). The combined organic layer was dried over anhydrous MgSO_4_ and concentrated *in vacuo*. The residue was subjected to column chromatography (eluant: CH_2_Cl_2_) to yield the title compound as colourless needles (yield: 956 mg, 65%; m.p. 348 – 349 K). IR (*ν*_max_, KBr, cm^−1^) 3356, 1630, 1444, 1291, 987, 976, 955, 747, 691; ^1^H NMR (400 MHz, CDCl_3_, *δ*_H_) 6.84 (1*H*, d, ^3^*J* = 5.6 Hz), 7.28 – 7.55 (6*H*, m), 7.94 (1*H*, t, ^3^*J* = 4.8 Hz); *δ*_C_ (100.5 MHz, CDCl_3_) 121.5 (CH), 127.0 (2 C, CH), 128.8 (2 C, CH), 129.0 (CH), 135.7 (C_quat_), 139.2 (CH), 152.0 (CH). Crystals for X-ray analysis were grown from a solution in CH_2_Cl_2_/hexane (1:1, *v*/*v*) by slow evaporation of the solvents.

### S3. Refinement

Crystal data, data collection and structure refinement details are summarized in Table 2. The OH H atoms were located in a difference Fourier map and freely refined. The C-bound H atoms were fixed geometrically (C—H = 0.95 Å) and allowed to ride on their parent atoms with *U*_iso_(H) = 1.2*U*_eq_(*C*).

### Crystal data

**Table:**

C_9_H_9_NO	*D*_x_ = 1.205 Mg m^−^^3^
*M**_r_* = 147.17	Melting point: 348 K
Orthorhombic, *P**b**c**a*	Mo *K*α radiation, λ = 0.71073 Å
*a* = 10.231 (5) Å	Cell parameters from 9623 reflections
*b* = 7.584 (3) Å	θ = 2.2–28.4°
*c* = 41.816 (18) Å	µ = 0.08 mm^−^^1^
*V* = 3245 (2) Å^3^	*T* = 100 K
*Z* = 16	Block, colourless
*F*(000) = 1248	0.2 × 0.2 × 0.1 mm

### Data collection

**Table:**

Bruker APEXII CCD diffractometer	3724 reflections with *I* > 2σ(*I*)
φ and ω scans	*R*_int_ = 0.022
Absorption correction: multi-scan (*SADABS*; Bruker, 2013)	θ_max_ = 28.6°, θ_min_ = 1.0°
*T*_min_ = 0.666, *T*_max_ = 0.746	*h* = −13→13
34431 measured reflections	*k* = −10→10
3944 independent reflections	*l* = −55→53

### Refinement

**Table:**

Refinement on *F*^2^	0 restraints
Least-squares matrix: full	Hydrogen site location: mixed
*R*[*F*^2^ > 2σ(*F*^2^)] = 0.043	H atoms treated by a mixture of independent and constrained refinement
*wR*(*F*^2^) = 0.113	*w* = 1/[σ^2^(*F*_o_^2^) + (0.0445*P*)^2^ + 2.1543*P*] where *P* = (*F*_o_^2^ + 2*F*_c_^2^)/3
*S* = 1.10	(Δ/σ)_max_ = 0.001
3944 reflections	Δρ_max_ = 0.45 e Å^−^^3^
207 parameters	Δρ_min_ = −0.19 e Å^−^^3^

### Special details

**Table:**

Geometry. All e.s.d.'s (except the e.s.d. in the dihedral angle between two l.s. planes) are estimated using the full covariance matrix. The cell e.s.d.'s are taken into account individually in the estimation of e.s.d.'s in distances, angles and torsion angles; correlations between e.s.d.'s in cell parameters are only used when they are defined by crystal symmetry. An approximate (isotropic) treatment of cell e.s.d.'s is used for estimating e.s.d.'s involving l.s. planes.

### Fractional atomic coordinates and isotropic or equivalent isotropic displacement parameters (Å^2^)

**Table:**

	*x*	*y*	*z*	*U*_iso_*/*U*_eq_	
O1B	0.27939 (9)	0.14918 (13)	0.83349 (2)	0.0214 (2)	
O1A	0.57897 (9)	0.36622 (12)	0.60376 (2)	0.0210 (2)	
N1A	0.65418 (10)	0.21056 (14)	0.60658 (2)	0.0172 (2)	
N1B	0.37727 (11)	0.02042 (14)	0.83755 (2)	0.0189 (2)	
C9A	0.66426 (11)	0.12592 (16)	0.57973 (3)	0.0162 (2)	
H9A	0.7096	0.0167	0.5804	0.019*	
C7A	0.65649 (11)	0.10018 (15)	0.52143 (3)	0.0146 (2)	
H7A	0.7135	0.0024	0.5242	0.017*	
C1A	0.62383 (11)	0.14952 (15)	0.48805 (3)	0.0137 (2)	
C6A	0.52038 (11)	0.26618 (15)	0.48030 (3)	0.0152 (2)	
H6A	0.4665	0.3124	0.4968	0.018*	
C2B	0.50012 (12)	−0.04774 (16)	0.69394 (3)	0.0171 (2)	
H2B	0.5770	−0.1114	0.6993	0.021*	
C5A	0.49730 (12)	0.31367 (16)	0.44838 (3)	0.0182 (2)	
H5A	0.4278	0.3920	0.4434	0.022*	
C7B	0.44406 (12)	−0.04211 (15)	0.75192 (3)	0.0167 (2)	
H7B	0.5154	−0.1202	0.7552	0.020*	
C2A	0.70079 (12)	0.08011 (17)	0.46297 (3)	0.0189 (2)	
H2A	0.7689	−0.0008	0.4678	0.023*	
C8A	0.61346 (11)	0.18013 (15)	0.54843 (3)	0.0152 (2)	
H8A	0.5502	0.2716	0.5471	0.018*	
C1B	0.41218 (11)	0.00325 (15)	0.71837 (3)	0.0153 (2)	
C3B	0.47534 (13)	−0.00548 (17)	0.66187 (3)	0.0202 (3)	
H3B	0.5352	−0.0408	0.6457	0.024*	
C5B	0.27317 (13)	0.13917 (17)	0.67760 (3)	0.0224 (3)	
H5B	0.1963	0.2023	0.6720	0.027*	
C6B	0.29760 (12)	0.09644 (16)	0.70966 (3)	0.0188 (2)	
H6B	0.2368	0.1303	0.7257	0.023*	
C4B	0.36242 (14)	0.08882 (17)	0.65358 (3)	0.0228 (3)	
H4B	0.3461	0.1186	0.6319	0.027*	
C8B	0.38138 (12)	0.01692 (16)	0.77854 (3)	0.0170 (2)	
H8B	0.3088	0.0942	0.7763	0.020*	
C9B	0.42381 (13)	−0.03653 (16)	0.81057 (3)	0.0191 (2)	
H9B	0.4921	−0.1212	0.8117	0.023*	
C4A	0.57625 (13)	0.24623 (18)	0.42372 (3)	0.0220 (3)	
H4A	0.5607	0.2799	0.4022	0.026*	
C3A	0.67797 (14)	0.12902 (18)	0.43106 (3)	0.0235 (3)	
H3A	0.7314	0.0828	0.4145	0.028*	
H1B	0.248 (2)	0.163 (2)	0.8539 (4)	0.039 (5)*	
H1A	0.588 (2)	0.415 (3)	0.6235 (5)	0.048 (6)*	

### Atomic displacement parameters (Å^2^)

**Table:**

	*U*^11^	*U*^22^	*U*^33^	*U*^12^	*U*^13^	*U*^23^
O1B	0.0216 (4)	0.0283 (5)	0.0144 (4)	0.0041 (4)	0.0037 (3)	0.0011 (3)
O1A	0.0301 (5)	0.0197 (4)	0.0131 (4)	0.0029 (4)	−0.0024 (3)	−0.0010 (3)
N1A	0.0181 (5)	0.0191 (5)	0.0144 (5)	−0.0022 (4)	−0.0008 (4)	0.0040 (4)
N1B	0.0237 (5)	0.0183 (5)	0.0146 (5)	−0.0013 (4)	−0.0001 (4)	0.0023 (4)
C9A	0.0169 (5)	0.0174 (5)	0.0141 (5)	−0.0026 (4)	−0.0012 (4)	0.0033 (4)
C7A	0.0143 (5)	0.0136 (5)	0.0159 (5)	−0.0011 (4)	−0.0030 (4)	0.0012 (4)
C1A	0.0158 (5)	0.0118 (5)	0.0134 (5)	−0.0026 (4)	−0.0024 (4)	−0.0012 (4)
C6A	0.0148 (5)	0.0147 (5)	0.0160 (5)	−0.0010 (4)	−0.0008 (4)	−0.0011 (4)
C2B	0.0167 (5)	0.0167 (5)	0.0178 (5)	−0.0018 (4)	0.0025 (4)	−0.0009 (4)
C5A	0.0179 (5)	0.0171 (5)	0.0195 (6)	−0.0008 (4)	−0.0054 (4)	0.0022 (4)
C7B	0.0196 (5)	0.0142 (5)	0.0164 (5)	0.0014 (4)	0.0010 (4)	0.0006 (4)
C2A	0.0206 (6)	0.0189 (6)	0.0172 (6)	0.0051 (5)	−0.0019 (4)	−0.0028 (4)
C8A	0.0164 (5)	0.0151 (5)	0.0141 (5)	−0.0020 (4)	−0.0023 (4)	0.0015 (4)
C1B	0.0189 (5)	0.0125 (5)	0.0144 (5)	−0.0018 (4)	0.0019 (4)	−0.0005 (4)
C3B	0.0254 (6)	0.0203 (6)	0.0149 (5)	−0.0065 (5)	0.0053 (5)	−0.0020 (4)
C5B	0.0265 (7)	0.0189 (6)	0.0218 (6)	0.0021 (5)	−0.0048 (5)	0.0013 (5)
C6B	0.0211 (6)	0.0179 (6)	0.0175 (6)	0.0020 (5)	0.0019 (4)	−0.0010 (4)
C4B	0.0324 (7)	0.0207 (6)	0.0152 (5)	−0.0065 (5)	−0.0032 (5)	0.0023 (5)
C8B	0.0206 (5)	0.0157 (5)	0.0146 (5)	−0.0005 (4)	0.0015 (4)	0.0009 (4)
C9B	0.0239 (6)	0.0171 (6)	0.0162 (5)	0.0001 (5)	0.0007 (4)	0.0022 (4)
C4A	0.0290 (6)	0.0236 (6)	0.0133 (5)	−0.0014 (5)	−0.0052 (5)	0.0011 (5)
C3A	0.0299 (7)	0.0270 (7)	0.0137 (5)	0.0033 (5)	0.0015 (5)	−0.0048 (5)

### Geometric parameters (Å, º)

**Table:**

C1A—C2A	1.4133 (17)	C6A—H6A	0.9500
C1A—C6A	1.4171 (16)	C6B—H6B	0.9500
C1B—C6B	1.4166 (17)	C7A—C8A	1.3548 (17)
C2A—C3A	1.4043 (18)	C7A—C1A	1.4834 (16)
C2A—H2A	0.9500	C7A—H7A	0.9500
C2B—C3B	1.4020 (17)	C7B—C8B	1.3606 (17)
C2B—C1B	1.4152 (16)	C7B—C1B	1.4806 (17)
C2B—H2B	0.9500	C7B—H7B	0.9500
C3A—H3A	0.9500	C8A—H8A	0.9500
C3B—C4B	1.402 (2)	C8B—C9B	1.4649 (17)
C3B—H3B	0.9500	C8B—H8B	0.9500
C4A—C3A	1.4027 (19)	C9A—C8A	1.4671 (16)
C4A—H4A	0.9500	C9A—H9A	0.9500
C4B—H4B	0.9500	C9B—H9B	0.9500
C5A—C4A	1.4063 (19)	N1A—C9A	1.2977 (16)
C5A—H5A	0.9500	N1B—C9B	1.2985 (16)
C5B—C4B	1.4100 (19)	O1A—H1A	0.91 (2)
C5B—C6B	1.4017 (18)	O1A—N1A	1.4141 (14)
C5B—H5B	0.9500	O1B—H1B	0.917 (19)
C6A—C5A	1.4026 (17)	O1B—N1B	1.4090 (14)
			
C1A—C2A—H2A	119.5	C5B—C4B—H4B	120.1
C1A—C6A—H6A	119.9	C5B—C6B—H6B	119.7
C1A—C7A—H7A	116.6	C5B—C6B—C1B	120.60 (11)
C1B—C6B—H6B	119.7	C6A—C5A—C4A	120.52 (11)
C1B—C7B—H7B	116.7	C6A—C5A—H5A	119.7
C1B—C2B—H2B	119.6	C6A—C1A—C7A	122.75 (10)
C2A—C3A—H3A	120.0	C6B—C5B—C4B	120.20 (12)
C2A—C1A—C6A	118.62 (11)	C6B—C5B—H5B	119.9
C2A—C1A—C7A	118.62 (11)	C6B—C1B—C7B	122.82 (10)
C2B—C3B—C4B	120.13 (11)	C7A—C8A—H8A	119.9
C2B—C3B—H3B	119.9	C7A—C8A—C9A	120.18 (11)
C2B—C1B—C6B	118.47 (11)	C7B—C8B—C9B	121.15 (12)
C2B—C1B—C7B	118.71 (11)	C7B—C8B—H8B	119.4
C3A—C4A—H4A	120.1	C8A—C7A—C1A	126.72 (11)
C3A—C4A—C5A	119.75 (11)	C8A—C7A—H7A	116.6
C3A—C2A—H2A	119.5	C8A—C9A—H9A	116.4
C3A—C2A—C1A	120.94 (11)	C8B—C9B—H9B	116.8
C3B—C4B—H4B	120.1	C8B—C7B—C1B	126.51 (11)
C3B—C4B—C5B	119.72 (12)	C8B—C7B—H7B	116.7
C3B—C2B—C1B	120.87 (12)	C9A—C8A—H8A	119.9
C3B—C2B—H2B	119.6	C9A—N1A—O1A	112.58 (9)
C4A—C3A—H3A	120.0	C9B—C8B—H8B	119.4
C4A—C3A—C2A	119.91 (11)	C9B—N1B—O1B	112.73 (10)
C4A—C5A—H5A	119.7	N1A—C9A—C8A	127.25 (11)
C4B—C5B—H5B	119.9	N1A—C9A—H9A	116.4
C4B—C3B—H3B	119.9	N1A—O1A—H1A	102.2 (13)
C5A—C4A—H4A	120.1	N1B—C9B—H9B	116.8
C5A—C6A—H6A	119.9	N1B—C9B—C8B	126.41 (12)
C5A—C6A—C1A	120.24 (11)	N1B—O1B—H1B	102.2 (12)
			
C1A—C2A—C3A—C4A	−1.0 (2)	C6A—C1A—C2A—C3A	1.73 (18)
C1A—C6A—C5A—C4A	0.04 (18)	C6B—C5B—C4B—C3B	0.50 (19)
C1A—C7A—C8A—C9A	−174.32 (11)	C7A—C1A—C2A—C3A	−177.00 (11)
C1B—C7B—C8B—C9B	−179.24 (11)	C7A—C1A—C6A—C5A	177.43 (11)
C1B—C2B—C3B—C4B	0.13 (18)	C7B—C8B—C9B—N1B	175.07 (12)
C2A—C1A—C6A—C5A	−1.24 (17)	C7B—C1B—C6B—C5B	178.75 (12)
C2B—C3B—C4B—C5B	−0.73 (19)	C8A—C7A—C1A—C2A	165.01 (12)
C2B—C1B—C6B—C5B	−0.92 (18)	C8A—C7A—C1A—C6A	−13.66 (18)
C3B—C2B—C1B—C6B	0.69 (18)	C8B—C7B—C1B—C6B	−9.56 (19)
C3B—C2B—C1B—C7B	−179.00 (11)	C8B—C7B—C1B—C2B	170.11 (12)
C4B—C5B—C6B—C1B	0.34 (19)	N1A—C9A—C8A—C7A	164.78 (12)
C5A—C4A—C3A—C2A	−0.2 (2)	O1A—N1A—C9A—C8A	3.45 (17)
C6A—C5A—C4A—C3A	0.71 (19)	O1B—N1B—C9B—C8B	−1.80 (18)

### Hydrogen-bond geometry (Å, º)

Cg1 and Cg2 are the centroids of rings C1A–C6A and C1B–C6B, respectively.

**Table:**

*D*—H···*A*	*D*—H	H···*A*	*D*···*A*	*D*—H···*A*
O1*A*—H1*A*···N1*B*^i^	0.91 (2)	1.85 (2)	2.755 (2)	174 (2)
O1*B*—H1*B*···N1*A*^ii^	0.92 (2)	1.95 (2)	2.853 (2)	170 (2)
C2*A*—H2*A*···*Cg*1^iii^	0.95	2.70	3.563 (2)	151
C5*B*—H5*B*···*Cg*2^iv^	0.95	2.80	3.508 (2)	132
C9*B*—H9*B*···*Cg*2^v^	0.95	2.82	3.717 (2)	159

Symmetry codes: (i) −*x*+1, *y*+1/2, −*z*+3/2; (ii) *x*−1/2, *y*, −*z*+3/2; (iii) −*x*+3/2, *y*−1/2, *z*; (iv) −*x*+1/2, *y*−1/2, *z*; (v) −*x*+1, *y*−1/2, −*z*+3/2.
