# Supplementary figures and images for: Crystal structure of (1Z,2E)-cinnamaldehyde oxime
Source: Acta Crystallogr E Crystallogr Commun. 2015 Dec 16;71(Pt 12):o1063–4. doi: 10.1107/S2056989015023853 (PMC4719980; doi:10.1107/S2056989015023853)

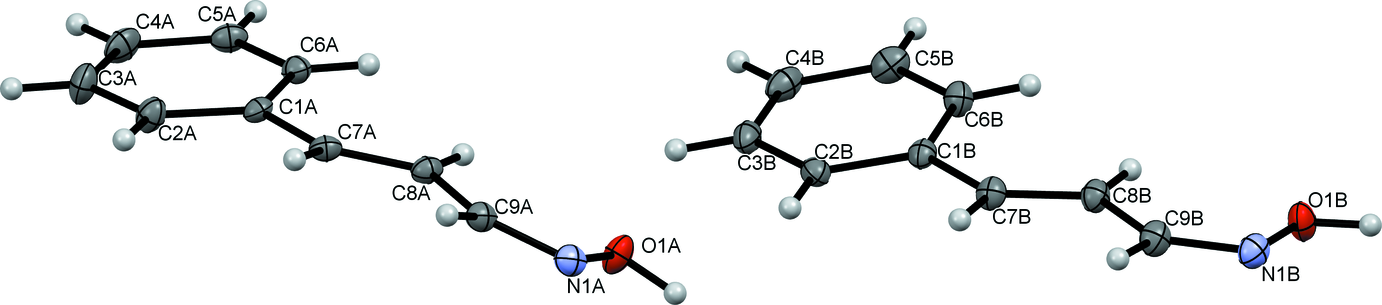

Supplement: Supplementary file 4 [file e-71-o1063-fig1.tif]

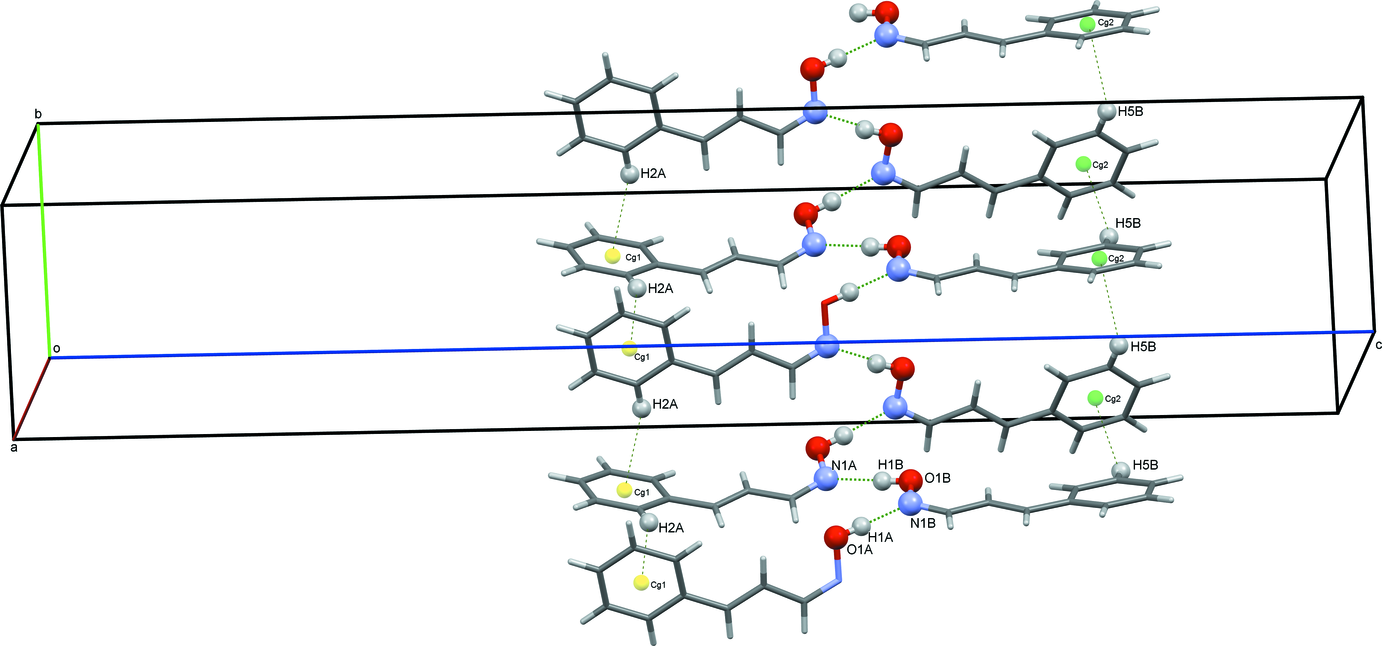

Supplement: Supplementary file 5 [file e-71-o1063-fig2.tif]

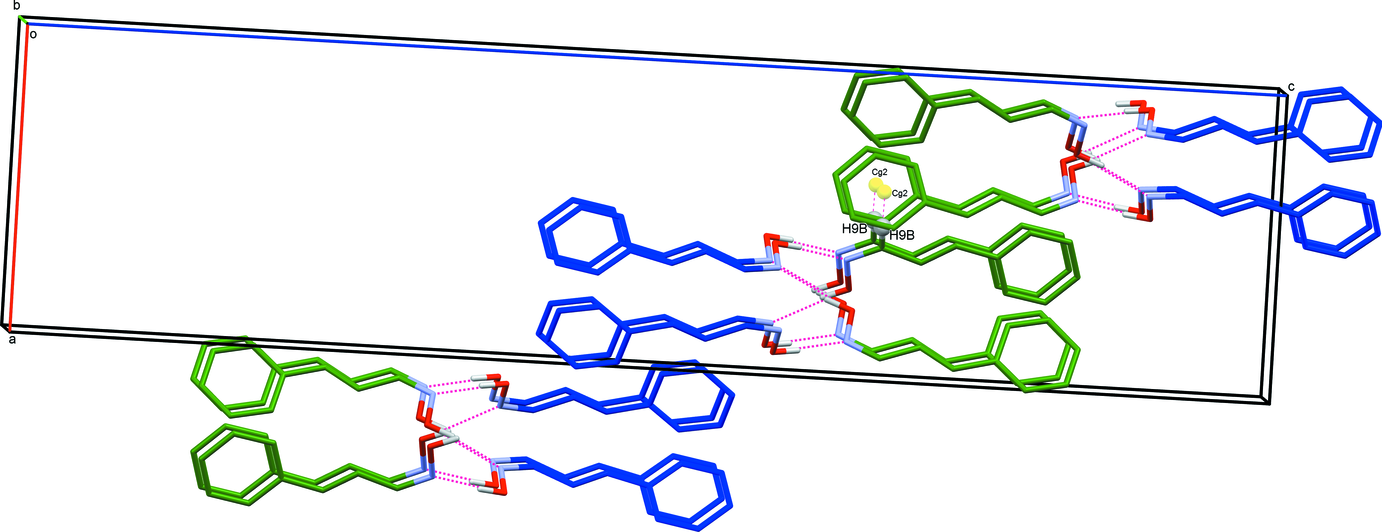

Supplement: Supplementary file 6 [file e-71-o1063-fig3.tif]
